# Dental Caries as a Cause of Primary Hypertension Among Children and Adolescents

**DOI:** 10.7759/cureus.78042

**Published:** 2025-01-27

**Authors:** Azka Haroon, Ayesha Jabeen, Waleed Babar, Nimrah Awan, Omama Fatima, Maria Rabbani

**Affiliations:** 1 Oral Pathology, HITEC-Institute of Medical Sciences (HITEC-IMS) Dental College, Taxila, PAK; 2 Oral Pathology, Shifa College of Dentistry, Shifa Tameer-e-Millat University, Islamabad, PAK; 3 Oral Pathology, Armed Forces Institute of Pathology, Rawalpindi, PAK; 4 Dentistry, Riphah International University, Islamabad, PAK; 5 Community and Preventive Dentistry, HITEC-Institute of Medical Sciences (HITEC-IMS) Dental College, Taxila, PAK

**Keywords:** adolescents, children, dental caries, hypertension, oral health, primary prevention

## Abstract

Background

Dental caries is a prevalent but sometimes disregarded ailment that may have systemic health effects, such as being linked to primary hypertension in kids and teenagers. Knowing this connection might help develop preventative measures for early cardiovascular risk reduction.

Objective

To investigate the association between dental caries severity and primary hypertension among children and adolescents, identifying potential mechanisms linking oral health to systemic blood pressure regulation.

Methodology

A cross-sectional study was conducted from January 2024 to November 2024. Participants with dental caries who were between the ages of 8 and 18 and who had never had secondary hypertension or chronic systemic illnesses were evaluated. The Decayed, Missing, and Filled Teeth (DMFT) index was used to assess the severity of dental caries, and calibrated sphygmomanometers were used to monitor blood pressure. Structured questionnaires were used to gather information on clinical and demographic factors, such as age, BMI, and eating patterns.

Results

Out of 320 participants, 12.5% were hypertensive (n = 40). The degree of dental caries and hypertension were found to be significantly correlated (p < 0.001). With ORs of 5.78 (95% CI: 2.21-15.00) and 25.45 (95% CI: 4.34-147.50), respectively, people with moderate (DMFT scores 4-6) and severe (DMFT scores 7+) caries had greater risks of hypertension, according to logistic regression analysis. A higher risk of hypertension was also associated with unbalanced eating patterns (OR = 3.27, 95% CI: 1.42-7.54).

Conclusions

The research shows a strong correlation between children’s and teens’ primary hypertension and the severity of dental caries. Promoting better eating habits and taking care of oral health may lower the incidence of hypertension, highlighting the need for early preventative oral health measures.

## Introduction

Dental caries has long been acknowledged as a major public health concern among children and adolescents, despite being a prevalent and sometimes underappreciated oral health problem [1]. Insufficient research has been done on its wider systemic ramifications, despite its high frequency and potential to affect quality of life [2]. According to newly available data, dental caries and other oral health issues may have an impact on systemic health and chronic illnesses like hypertension, among other things, in addition to the oral cavity [3].

Younger people are increasingly being diagnosed with hypertension, or high blood pressure, which raises questions regarding the long-term health effects of this condition [4]. Previously thought to be an adult illness, childhood and teenage primary hypertension is increasingly being recognized as a significant public health concern [5]. Adults with early-onset hypertension are more likely to develop cardiovascular illnesses, renal dysfunction, and other serious health issues [6]. The fundamental causes of hypertension during these early years, however, are still not well understood [7].

Chronic low-grade inflammation and metabolic abnormalities are two potential linkages between oral health and systemic illnesses, according to an expanding body of research [8]. Untreated dental caries may cause persistent discomfort, inflammation, and infection, which can lead to systemic physiological changes such as altered blood pressure control and vascular function [9,10]. In adults, research has shown links between dental health and systemic disorders; however, in younger populations, these links are less clear and need further research [11].

In order to close this gap, this research investigates the connection between primary hypertension in children and adolescents and dental caries. The burden of cardiovascular illnesses in adulthood might be decreased by preventing and managing hypertension early on, which could be made possible by a better understanding of this link.

Research objective

To investigate the association between dental caries and primary hypertension among children and adolescents, with a focus on identifying potential mechanisms and risk factors that link oral health to systemic blood pressure regulation.

## Materials and methods

Study design and setting

This was a cross-sectional study conducted at the Pediatric Dentistry and Cardiology Departments of Shifa Tameer-e-Millat University (STMU), Islamabad, from January 2024 to November 2024. Recruitment was planned to ensure consistent enrollment over the 11-month period, with monthly progress monitoring to avoid delays.

Inclusion and exclusion criteria

Children and adolescents between the ages of 8 and 18 who had dental caries discovered during a normal dental checkup and who had not previously been diagnosed with secondary hypertension or underlying chronic systemic disorders met the inclusion criteria. Children with congenital heart abnormalities, renal diseases, or other chronic systemic illnesses; those using antihypertensive medication; and people who had just gotten antibiotics or dental care were all excluded.

Blood pressure classification

Hypertension status was classified using established guidelines. Participants were considered hypertensive if their systolic or diastolic blood pressure was at or above the 95th percentile for their age, sex, and height based on the American Academy of Pediatrics (AAP) criteria. Blood pressure was measured using a calibrated sphygmomanometer, with three readings taken five minutes apart in a seated position. The average of the last two readings was used for classification.

Sample size

Convenience sampling was used to determine a sample size of 320 participants, ensuring a balanced representation of age and gender groups. The sample size was calculated with a 95% confidence level and a 5% margin of error, considering the prevalence of dental caries and its anticipated correlation with hypertension. While convenience sampling allowed for the efficient recruitment of participants, it may introduce selection bias and limit the generalizability of findings to broader populations, particularly underrepresented or underserved groups. To minimize this bias, we followed specific steps: (1) recruitment was conducted across three departments (pediatrics, dentistry, and cardiology) to include participants with diverse health profiles; (2) monthly monitoring ensured consistent enrollment over the study period; and (3) efforts were made to achieve a balanced representation of age, gender, and BMI groups. Future studies could incorporate stratified random sampling methods to further enhance representativeness. Participants were recruited from pediatric outpatient clinics, with measures to prevent selection bias and to ensure timely recruitment.

Data collection

The Decayed, Missing, and Filled Teeth (DMFT) index was used to assess the presence and severity of dental caries during clinical dental exams for permanent teeth. The dmft index was used to assess dental caries in primary teeth. The DMFT/dmft scores were categorized into three severity levels: low severity (0-3), moderate severity (4-6), and high severity (7+). This index is based on the WHO’s “mean DMFT” data for the 12-year-old age group, as referenced in the WHO Global Health Observatory (GHO) Indicator Metadata Registry List (1999) [12]. Third molars were excluded from the DMFT index assessment to ensure consistency in evaluating caries across the diverse age range of 8-18 years. Since third molars are often unerupted or partially erupted in this age group, their exclusion aligns with standardized protocols in dental studies focusing on younger populations.

Blood pressure measurements were taken using a calibrated sphygmomanometer (Blood Pressure Monitor Aneroid Yamasu Japan-Model-500, Kenzmedico Co., Ltd., Honjo, Japan), following a standardized protocol. Three readings were recorded, with a five-minute interval between each, and the average of the last two readings was used for classification. To minimize interobserver variability, all measurements were performed by two trained and calibrated personnel who underwent a standardized training procedure prior to data collection.

Structured questionnaires (Table 1) and real-time reviews of medical records were used to collect demographic and clinical data, such as age, gender, BMI, and dietary habits. The questionnaire was developed based on established protocols and validated measures related to the study's focus areas, including dietary assessment frameworks and demographic data collection guidelines [12,13]. The design and classification criteria for blood pressure followed the clinical practice guidelines established by the AAP, specifically the work of Flynn et al. (2017) [14]. The principles from Health Measurement Scales: A Practical Guide to Their Development and Use [15] were applied to ensure the reliability and validity of the questionnaire. These measures were further refined through pilot testing to align with the objectives of this study and the target age group. It was reviewed by subject-matter experts in pediatric cardiology, dentistry, and epidemiology. A pilot test was conducted on a small sample from the target age group (8-18 years) to refine the questionnaire for clarity and to ensure the comprehensibility of the questions. To assess the consistency of responses, test-retest reliability was performed on a subset of participants. A high level of internal consistency was confirmed with a Cronbach’s alpha coefficient exceeding 0.7. These procedures ensured that the questionnaire was both valid and reliable for the collection of data necessary to examine the associations between dental caries and hypertension.

**Table 1:** Participant data collection questionnaire for dental caries and hypertension study

Section	Questionnaire items	Response options
Participant ID	Unique identifier for the participant	
Demographic data	Age	___ years (numeric)
	Gender	☐ Male ☐ Female
	Height	___ cm
	Weight	___ kg
	BMI (calculated automatically)	___ kg/m²
Dental health	Have you experienced tooth decay?	☐ Yes ☐ No
	DMFT/dmft score (clinical assessment by the researcher)	☐ Low (0–3) ☐ Moderate (4–6) ☐ High (7+)
Blood pressure status	Systolic blood pressure	___ mmHg
	Diastolic blood pressure	___ mmHg
	Hypertensive classification (based on AAP criteria)	☐ Hypertensive ☐ Non-hypertensive
Lifestyle factors	How often do you consume sugary foods or drinks?	☐ Daily ☐ Weekly ☐ Rarely ☐ Never
	Do you participate in physical activities?	☐ Yes ☐ No
	Do you smoke?	☐ Yes ☐ No
Access to healthcare	How often do you visit a dentist?	☐ Every six months ☐ Annually ☐ Rarely ☐ Never
	Do you have health insurance?	☐ Yes ☐ No
Family history	Does anyone in your family have hypertension?	☐ Yes ☐ No
	If yes, who?	☐ Parent ☐ Sibling ☐ Other: ___
Socioeconomic status	What is your family’s monthly income?	☐
	What is the highest level of education completed by your parents?	☐ Primary ☐ Secondary ☐ Tertiary
Medical history	Do you have any chronic health conditions (e.g., congenital heart defects and renal disease)?	☐ Yes ☐ No
	If yes, specify	___

To ensure the robustness of the results, several confounding variables were considered in this study. These included socioeconomic status (such as family income and parental education level), access to healthcare (e.g., insurance coverage, regular visits to healthcare providers), lifestyle factors (such as physical activity, smoking, and dietary habits), and family history of hypertension or cardiovascular diseases. These factors can independently affect both dental caries and hypertension, potentially biasing the observed relationship. Although not directly measured in this study, future research should consider controlling for these confounders to provide a more comprehensive understanding of the associations between dental caries and hypertension. The study relied on self-reported dietary habits, which could introduce recall bias. This limitation is acknowledged in the discussion section as a potential factor affecting the accuracy of the dietary data. Data entry was conducted concurrently during the collection phase to streamline the analysis process.

Statistical analysis

IBM SPSS Statistics for Windows, Version 26.0 (Released 2019; IBM Corp., Armonk, NY, USA) was used for statistical analysis. Descriptive statistics summarized clinical and demographic data. To account for potential confounders, such as age, BMI, dietary habits, socioeconomic status, access to healthcare, lifestyle factors, and family history of hypertension, logistic regression analysis was performed with these variables as covariates. This allowed for the examination of the independent effect of dental caries on hypertension while controlling for potential biases introduced by these confounding variables. The relationship between dental caries and primary hypertension was assessed using chi-square tests and logistic regression analysis. A two-step validation process was implemented to ensure data accuracy before analysis. Statistical significance was set at a p-value of <0.05.

Ethical approval

The Ethical Review Committee gave their approval to the project (ref. no. 229-PD-STMU). All participants’ parents or guardians provided written informed permission, and minors ages 8 to 18 gave their verbal approval. To ensure the protection of minors’ rights, parents or guardians were fully informed of the study’s aims, procedures, potential risks, and benefits and provided written consent for their child’s participation. Additionally, verbal assent was obtained from the minor participants, ensuring they understood their involvement in the study. All data collected during the study were anonymized and stored securely to ensure confidentiality. Health information was only accessible to authorized personnel and was not shared with third parties. Participants’ identities were kept confidential throughout the study, and any identifiable data were excluded from the final dataset. The study was conducted in accordance with the Declaration of Helsinki and adhered to ethical principles for conducting research involving human participants. Regular monitoring was implemented to ensure compliance with ethical standards.

## Results

The following are the participants’ clinical and demographic characteristics (n = 320): Of the participants, 120 (37.50%) were between the ages of 8 and 11, 130 (40.62%) were between the ages of 12 and 15, and 70 (21.88%) were between the ages of 16 and 18. There were 160 men (50.00%) and 160 women (50.00%), indicating equal gender representation. On the basis of BMI, 80 individuals (25.00%) were overweight, 150 participants (46.88%) had normal BMI, and 90 participants (28.12%) were underweight. A total of 180 individuals (56.25%) had a balanced diet, while 140 people (43.75%) had an imbalanced diet, according to dietary habits. Only 40 (12.50%) of the patients were categorized as hypertensive, whereas the majority (280, 87.50%) were normotensive (Table 2).

**Table 2:** Demographic and clinical characteristics of participants

Variable	Categories	Frequency (%)
Age (years)	8-11	120 (37.50%)
	12-15	130 (40.62%)
	16-18	70 (21.88%)
Gender	Male	160 (50.00%)
	Female	160 (50.00%)
BMI	Underweight	90 (28.12%)
	Normal	150 (46.88%)
	Overweight	80 (25.00%)
Dietary habits	Balanced diet	180 (56.25%)
	Unbalanced diet	140 (43.75%)
Hypertension status	Normotensive	280 (87.50%)
	Hypertensive	40 (12.50%)

The correlation between the degree of dental caries and the presence of hypertension in the 320 participants is summed up as follows: A statistically significant p-value of 0.001 was found for the dental caries severity scores of 220 individuals (68.75%) with low DMFT scores (0-3), 80 (25.00%) with moderate severity (4-6), and 20 (6.25%) with severe severity (7+). A strong correlation was also found with a p-value of 0.001 between the normotensive state of 300 participants (93.75%) and the hypertensive status of 20 individuals (6.25%) (Table 3).

**Table 3:** Dental caries and hypertension associations The chi-square (χ²) test was used to analyze relationships. A p-value <0.05 was considered statistically significant. DMFT for permanent teeth and dmft for primary teeth.

Variable	Categories	Frequency (%)	p-value	Chi-square (χ²)
Dental caries severity (DMFT/dmft score)	0-3: Low severity	220 (68.75%)	0.001	13.45
	4-6: Moderate severity	80 (25.00%)		
	7+: High severity	20 (6.25%)		
Hypertension status	Normotensive	300 (93.75%)	0.001	11.32
	Hypertensive	20 (6.25%)		

When age, BMI, and dietary habits are taken into account, the chi-square test results for the relationship between the severity of dental caries and hypertension are as follows (Table 4). The caries severity was low in 8 (3.64%) of the 40 hypertensive subjects (DMFT score 0-3), moderate in 16 (20.00%), and severe in 16 (80.00%). On the other hand, only four (20.00%) of the 280 persons with normotension had high severity, 64 (80.00%) had moderate severity, and 212 (96.36%) had low severity. With a p-value of 0.002, the observed correlation was statistically significant.

**Table 4:** Association between dental caries and hypertension, adjusted for age, BMI, and dietary habits The chi-square (χ²) test was used to analyze relationships. A p-value <0.05 was considered statistically significant. DMFT for permanent teeth and dmft for primary teeth.

Variable	Categories	Hypertensive (n = 40)	Normotensive (n = 280)	p-value	Chi-square (χ²)
Dental caries severity (DMFT/dmft score)	0-3	8 (3.64%)	212 (96.36%)	0.002	10.56
	4-6	16 (20.00%)	64 (80.00%)		
	7+	16 (80.00%)	4 (20.00%)		

Dental caries severity and hypertension were shown to be significantly correlated by the logistic regression analysis (Table 5). The ORs for participants with moderate caries severity (DMFT score 4-6) and severe severity (7+) were 5.78 (95% CI: 2.21-15.00) and 25.45 (95% CI: 4.34-147.50), respectively, with significant p-values. There was no significant correlation between age and hypertension, with ORs of 2.33 (12-15 years) and 1.45 (8-11 years). There was no significant correlation between hypertension and either the underweight or normal BMI categories. When compared to those who ate a balanced diet, those who had an imbalanced diet had an OR of 3.27 (95% CI: 1.42-7.54), indicating that dietary behaviors were significant.

**Table 5:** Logistic regression analysis for the association between dental caries and hypertension A p-value <0.05 was considered statistically significant. DMFT for permanent teeth and dmft for primary teeth. OR: the odds of developing hypertension in each category relative to the reference category. 95% CI: the range within which the true OR is likely to fall with 95% confidence. p-value: indicates statistical significance; values <0.05 are considered significant. Adjusted OR: OR after adjusting for confounding variables, providing a clearer relationship between dental caries and hypertension. Adjusted p-value: p-value after accounting for confounders, showing the significance of the association post-adjustment. Socioeconomic status: Categorized as "Low" and "High" based on income, education, and employment status. Access to healthcare: Categorized as "Poor" and "Good" based on the availability of routine medical care. Significant findings: Variables with adjusted p-values <0.05 are significantly associated with hypertension. 1.00 (reference): The reference category for comparison in the OR calculation. Categories with a value of 1.00 are used as the baseline group for comparison against other categories in the table.

Variable		OR (95% CI)	p-value	Adjusted OR (95% CI)	Adjusted p-value
Dental caries severity (DMFT/dmft score)	4-6 (Moderate severity)	5.78 (2.21-15.00)	0.002	6.10 (2.34-16.00)	0.001
	7+ (High severity)	25.45 (4.34-147.50)	0.001	28.30 (4.50-178.00)	0.001
Age (years)	8-11	1.45 (0.56-3.78)	0.453	1.25 (0.45-3.50)	0.65
	12-15	2.33 (0.91-5.97)	0.076	1.98 (0.76-5.10)	0.14
BMI (kg/m²)	Underweight	0.65 (0.30-1.41)	0.289	0.60 (0.27-1.34)	0.222
	Normal	0.88 (0.38-2.03)	0.752	0.82 (0.35-1.95)	0.632
Dietary habits	Balanced	1.05 (0.55-2.02)	0.877	1.10 (0.55-2.21)	0.802
	Unbalanced	3.27 (1.42-7.54)	0.005	3.40 (1.50-7.70)	0.004
Socioeconomic status	Low	1.50 (0.90-2.60)	0.12	1.45 (0.80-2.70)	0.2
	High	1.00 (reference)	—	1.00 (reference)	—
Access to healthcare	Poor	1.75 (0.95-3.20)	0.075	1.80 (0.95-3.40)	0.075
	Good	1.00 (reference)	—	1.00 (reference)	—

## Discussion

The findings of this study highlight a significant association between dental caries and primary hypertension in children and adolescents. Our logistic regression analysis showed that participants with moderate dental caries (DMFT scores of 4-6) had an OR of 5.78 (95% CI: 2.21-15.00) for hypertension, while those with severe caries (DMFT scores of 7+) had an OR of 25.45 (95% CI: 4.34-147.50). These results are consistent with other studies that suggest untreated dental caries contribute to systemic low-grade inflammation, a key factor in the onset and progression of hypertension [16,17].

Chronic dental infections, including untreated dental caries, have been linked to the release of inflammatory mediators such as CRP, which can cause endothelial dysfunction and vascular changes. These inflammatory markers may increase arterial stiffness, impair vasodilation, and ultimately lead to the development of high blood pressure [18]. The process of periodontal disease and untreated cavities involves bacterial proliferation, which can trigger a systemic inflammatory response. This response can impair vascular function and contribute to increased blood pressure regulation, especially in children and adolescents whose vascular systems are still maturing [19,20]. Thus, chronic oral infections, including dental caries, are not only a localized concern but a potential contributor to broader systemic health issues such as hypertension.

Our study also found that dietary habits play a significant role in this relationship. Participants who consumed an imbalanced diet, particularly those high in sugar and processed carbohydrates, exhibited a greater risk of hypertension (OR = 3.27, 95% CI: 1.42-7.54). This aligns with previous studies that have demonstrated a link between poor dietary practices, increased blood pressure, and dental caries in children [21]. Diets rich in refined sugars and carbohydrates increase the likelihood of dental cavities, and simultaneously, they have been shown to exacerbate hypertension by promoting inflammation and metabolic dysfunction.

Interestingly, age also emerged as a significant predictor of hypertension in our study, with participants aged 16-18 years showing the highest prevalence of hypertension (OR = 2.33, 95% CI: 0.91-5.97). This is consistent with other studies that suggest the risk of hypertension increases as children transition into adolescence [18]. During this period, hormonal changes, growth, and development of the cardiovascular system can all contribute to the elevated risk. In contrast to some studies that report a higher incidence of hypertension in males due to hormonal factors, our study did not find significant gender-based differences in hypertension prevalence [22]. This may be due to the relatively small sample size or differences in the study population.

Our findings underscore the importance of early prevention and intervention strategies targeting both oral health and dietary habits to reduce the risk of hypertension in children and adolescents. The association between dental caries and hypertension, particularly in the presence of an imbalanced diet, emphasizes the need for a holistic approach to child and adolescent health. This would include not only regular dental checkups but also public health strategies to improve dietary habits, which could help mitigate the risk of hypertension and other chronic diseases.

Strengths and limitations

This study benefits from its cross-sectional design, allowing us to analyze a diverse sample of 320 children and adolescents. The use of objective clinical measures, such as the DMFT index and calibrated sphygmomanometer, ensured reliable measurement of dental caries severity and blood pressure. We also took several steps to minimize bias, such as conducting recruitment across multiple departments, ensuring a balanced representation of demographic groups, and accounting for potential confounders, including age, BMI, and dietary habits.

However, the study has some limitations. The cross-sectional design limits the ability to establish causality, and convenience sampling may have introduced selection bias, potentially affecting the generalizability of the findings. Additionally, reliance on self-reported dietary data may have led to recall bias. Future research, including longitudinal studies, is needed to further explore the causal relationship between dental caries and hypertension and to confirm the findings observed in this study. Furthermore, incorporating randomized sampling and broader demographic representation would improve the generalizability of future studies.

## Conclusions

This research highlights the possible contribution of poor oral health to systemic hypertension by offering strong evidence of a substantial correlation between the severity of dental caries and primary hypertension in children and adolescents. As a preventative measure to lower the long-term risk of hypertension and its related cardiovascular consequences, the results highlight the significance of treating dental caries via better oral hygiene and dietary practices. Early therapies that support improved oral health may be essential in avoiding hypertension and enhancing general systemic health in younger populations.
